# Nucleus Accumbens Dopamine Levels Fluctuate Across Different States of Consciousness Under Sevoflurane Anesthesia

**DOI:** 10.3390/brainsci15090897

**Published:** 2025-08-22

**Authors:** Weiwei Bao, Fangjiaqi Wei, Jian Huang, Zhili Huang, Changhong Miao

**Affiliations:** 1 Department of Anesthesiology, Zhongshan Hospital, Fudan University, Shanghai 200032, China; bao.weiwei@zs-hospital.sh.cn (W.B.); 23211210082@m.fudan.edu.cn (F.W.); huang.jian1@zs-hospital.sh.cn (J.H.); huangzl@fudan.edu.cn (Z.H.); 2 State Key Laboratory of Brain Function and Brain Disorders and MOE Frontiers Centre for Brain Science, Department of Pharmacology, School of Basic Medical Sciences, Institutes of Brain Science, Joint International Research Laboratory of Sleep, Shanghai Medical College, Fudan University, Shanghai 200032, China

**Keywords:** dopamine, neurotransmitter, nucleus accumbens, sevoflurane anesthesia

## Abstract

Background: Dopamine (DA) is a critical neurotransmitter that regulates many physiological and behavioral processes. The central dopaminergic system plays a pivotal role in modulating general anesthesia (GA). DA release in the brain is mainly concentrated in the nucleus accumbens (NAc), prefrontal cortex, hypothalamus, and dorsal striatum. Several NAc neuron subtypes are essential for modulating states of consciousness during GA. However, whether NAc DA signal dynamics correlate with different states of consciousness under sevoflurane anesthesia remains to be elucidated. In this study, we measured the dynamic fluctuations of NAc DA levels throughout sevoflurane anesthesia to verify its role. Methods: An intensity-based genetically encoded DA indicator, dLight1.1, was employed to track DA release in the NAc. Fiber photometry combined with electroencephalogram/electromyogram recordings was employed to synchronously track NAc DA signal dynamics across different states of consciousness under sevoflurane anesthesia. Results: Under 2.5% sevoflurane exposure, DA release in the NAc significantly increased during the initial 100 s of sevoflurane induction, which was designated as sevo on-1 (mean ± standard error of the mean [SEM]; baseline vs. sevo on-1, *p* = 0.0261), and continued to decrease in the subsequent anesthesia maintenance phases (sevo on-1 vs. sevo on-4, *p* = 0.0070). Following the cessation of sevoflurane administration (with intervals denoted as sevooff), NAc DA gradually returned to baseline levels (sevo on-1 vs. sevo off-1, *p* = 0.0096; sevo on-1 vs. sevo off-3, *p* = 0.0490; sevo on-1 vs. sevo off-4, *p* = 0.0059; sevo on-4 vs. sevo off-4, *p* = 0.0340; sevo off-1 vs. sevo off-4, *p* = 0.0451). During the induction phase, NAc DA signal dynamics markedly increased during the pre-loss of consciousness (LOC) period (pre-anesthesia baseline vs. pre-LOC, *p* = 0.0329) and significantly declined after LOC (pre-LOC vs. post-LOC, *p* = 0.0094). For the emergence period, NAc DA release exhibited a noticeable increase during the initial period after recovery of consciousness (ROC) (anesthesia baseline vs. post-ROC, *p* = 0.0103; pre-ROC vs. post-ROC, *p* = 0.0086). Furthermore, the DA signals peaked rapidly upon the initiation of the burst wave and then gradually attenuated, indicating a positive correlation with the burst wave onset during burst suppression events. Conclusions: Our findings revealed that NAc DA neurotransmitter signal dynamics correlate with different states of consciousness throughout sevoflurane anesthesia.

## 1. Introduction

Sevoflurane has become a widely used inhalation anesthetic in clinical anesthesia owing to its rapid onset, stable metabolism, minimal respiratory irritation, and less environmental effects [1,2,3,4]. However, despite its well-established clinical use, the exact neural mechanisms of sevoflurane general anesthesia (GA), particularly in terms of the role of neurotransmitter fluctuations across consciousness transitions under GA, remain unclear [3,5,6].

Previous studies have demonstrated that the central dopaminergic (DAergic) system plays a crucial role in GA [3]. Dopamine (DA) is implicated in many neurological functions, including movement, cognition, reward, and arousal [7,8]. Additionally, DA release has been reported to be concentrated in the nucleus accumbens (NAc), dorsal striatum, medial prefrontal cortex, and hypothalamus [8,9]. However, the dynamically fluctuating DA release in specific nuclei and its associations with consciousness in GA are poorly understood.

The NAc, a key component of the ventral striatum, serves as a vital hub for DAergic transmission. The NAc is characterized by high levels of DA receptor-expressing neurons and receives mesolimbic DAergic afferents from the ventral tegmental area [10]. NAc DA release has also been reported to be elicited by NAc cholinergic interneurons and the basolateral amygdala to drive motivated behavior [11,12]. In addition, recent studies have revealed that several subtypes of NAc neurons are essential for modulating GA [13,14,15,16]. These studies suggest that dynamics of DA neurotransmitter signal in the NAc may be involved in regulating states of consciousness under GA. However, the precise characteristics and correlations of NAc DA fluctuations throughout the consciousness transition under sevoflurane GA have not yet been explored.

In the present study, we measured the NAc DA signal dynamics of male C57BL/6J mice during sevoflurane anesthesia through the in vivo real-time monitoring of a genetically encoded DA sensor called dLight1.1. Taken together, our results provide strong evidence that the dynamical release of NAc DA neurotransmitters is correlated with states of consciousness under sevoflurane anesthesia.

## 2. Materials and Methods

### 2.1. Animals

C57BL/6J mice (male, 8–10 weeks old, 22–28 g) were obtained from the Shanghai Laboratory Animal Center, Chinese Academy of Sciences (SLAC, Shanghai, China). The mice were housed in groups at a constant temperature (22 ± 0.5 °C) and humidity (55% ± 5%), under an automatic 12 h/12 h light/dark cycle (lights on at 07:00 and off at 19:00; illumination intensity ≈ 100 lux) [8,13,14,17]. Food and water were provided ad libitum. All efforts were made to minimize animal suffering and the number of animals used to generate reliable data. All animal procedures were conducted in accordance with the guidelines of the Care and Use Committee of Laboratory Animals of Fudan University.

### 2.2. Surgery

To detect dynamic changes in the DA levels in the NAc, we employed a DA indicator rAAV-CAG-dLight1.1-WPRE-hGH polyA (PT-1138; BrainVTA, Wuhan, China) [8]. Adult male mice were anesthetized with 1–2% isoflurane and fixed on a stereotaxic apparatus (RWD Life Science, Shenzhen, China). After shaving the fur on the head and sterilizing the skin with 75% ethanol, an incision was made to reveal the skull, and a minimum perforation was conducted on the exposed skull surface. A total of 70 nL AAV-dLight1.1 with titer 3–4 × 10^12^ vg/mL was unilaterally microinjected into the NAc (anteroposterior [AP]: +1.2 mm; mediolateral [ML]: −1.0 mm; dorsoventral [DV]: −4.5 mm) of mice through a glass pipette over 5 min via nitrogen gas pulses of 20–40 psi delivered by an air compression system (Picospritzer III, Parker Hannifin Corp, Cleveland, OH, USA) as described previously [8,13,14]. The pipette was then retained for 10 min, after which it was slowly pulled out. The incision was sutured using a medical surgical suture needle. The mice were then placed on a hot blanket of 37 °C until they woke up. After 3 weeks of postoperative recovery and viral expression, the mice were unilaterally implanted with an optical fiber cannula (125 mm outer diameter [OD], 0.37 numerical aperture [NA]; Newdoon, Hangzhou, China) above the NAc (AP: +1.2 mm; ML: −1.0 mm; DV: −4.0 mm), followed by electroencephalogram/electromyogram (EEG/EMG) electrode implantation, as previously described [13]. Behavioral experiments were conducted 1–2 weeks after surgery.

### 2.3. Fiber Photometry with EEG/EMG Recordings and Analysis

To detect dLight1.1 signals in the NAc during consciousness transition under sevoflurane anesthesia, we utilized a fiber photometry system (Thinkerbiotech, Nanjing, China) combined with EEG/EMG recordings, using previously described methods [8,17]. To record fluorescence signals, the beam from a 488 nm laser (OBIS 488LS, Coherent, Santa Clara, CA, USA) was reflected by a dichroic mirror (MD498; Thorlabs, Newton, NJ, USA), focused by a 10× objective lens (NA = 0.3, Olympus, Tokyo, Japan), and then coupled to an optical commutator (Doric Lenses, Canada). An optical fiber patch cord guided the light between the commutator and the implanted optical fiber. The laser power at the optical fiber tip was adjusted to a low level of 10–20 µW to minimize bleaching. The dLight1.1 fluorescent signal was bandpass-filtered (MF525-39, Thorlabs, Newton, NJ, USA) and collected using a photomultiplier tube (R3896, Hamamatsu, Shizuoka, Japan). An amplifier (C7319, Hamamatsu, Shizuoka, Japan) was used to convert the current output of the photomultiplier tube into voltage signals, which were further filtered through a low-pass filter (40 Hz cut-off; Brownlee 440). The photometry analog voltage signals were digitalized at 512 Hz and recorded synchronously with EEG/EMG recordings using a Power 1401 digitizer and Spike2 software (Version 8.11b, CED, Cambridge, UK).

An online randomization tool (https://www.random.org/lists/, accessed on 26 February 2024) was used to assign animals to different treatment groups (1.5%, 2.0%, or 2.5% sevoflurane). The mice were placed in a glass chamber to acclimatize to the environment, after which the dLight1.1 and EEG/EMG signals were simultaneously recorded [8,13,17]. They were exposed to 1–2 L/min 100% oxygen for 10 min to establish baseline values. Subsequently, sevoflurane (1.5%, 2.0%, or 2.5%) with oxygen was delivered via a sevoflurane vaporizer (VP 300; Beijing Aeonmed, Beijing, China). The sevoflurane concentrations were continuously monitored using an anesthesia monitor (IntelliVue MP20, Royal Dutch Philips Electronics, Amsterdam, The Netherlands) connected to the chamber. After 20 min of anesthesia, the sevoflurane vaporizer was turned off, and oxygen was still supplied. The recordings were continued for another 20 min after sevoflurane was turned off. In total, we recorded dLight1.1 signals for 50 min (10 min before, 20 min during, and 20 min after sevoflurane administration).

To conduct a detailed analysis of NAc DA release in the induction and emergence periods of sevoflurane anesthesia, the entire process was segmented into nine distinct time periods, ranging from −10 to 40 min, with 0 s marking the onset of sevoflurane administration. Specifically, we defined the awake baseline as 10 min prior to anesthesia (−10–0 min) and split the 20 min (0–20 min) sevoflurane inhalation into four time periods: sevo on-1 for 0–100 s; sevo on-2 for 100–200 s; sevo on-3 for 200–300 s; and sevo on-4 for 300–1200 s. Likewise, the duration following the recovery period of anesthesia (20–40 min) was also separated into four time intervals: sevo off-1 for 1200–1300 s; sevo off-2 for 1300–1400 s; sevo off-3 for 1400–1500 s; and sevo off-4 for 1500–2400 s.

We further examined the real-time NAc dLight1.1 signal dynamics during the induction (from wakefulness to the LOC state) and emergence (from anesthesia to the ROC state) phases of 2.5% sevoflurane GA, as defined by EEG/EMG recordings [13,18]. We identified state transitions and matched ΔF/F within a time frame of −300 s to +600 s before and after administration of 2.5% sevoflurane. LOC onset was defined as the transition from a low-amplitude, high-frequency EEG to a high-amplitude, low-frequency EEG, accompanied by continuous muscle tone minimization. Changes in the dLight1.1 signals during sevoflurane induction were analyzed in three continuous time segments (from −300 s to 600 s; 0 s represents the onset of 2.5% sevoflurane): pre-anesthesia baseline (from −300 s to 0 s); the early period of anesthesia before LOC (pre-LOC, from 0 s to LOC); and the initial period after LOC (post-LOC, from LOC to 600 s). Moreover, the start of ROC was identified as the moment when a low-amplitude, high-frequency EEG was combined with a significant increase in EMG activity. We analyzed the dLight1.1 signals during the sevoflurane emergence phase within three consecutive time sections (from −300 s to 600 s, and 0 s was the time point of 2.5% sevoflurane turning off): unconsciousness anesthesia baseline (from −300 s to 0 s); the pre-ROC period (from 0 s to ROC); and the initial post-ROC period (from ROC to 600 s). The dLight1.1 signal data recorded in Spike2 software (Version 8.11b, CED, Cambridge, UK) were transformed to MATLAB format and further analyzed using MATLAB software (R2023b, Mathworks, Natick, MA, USA) [8,13,17]. The photometry signal, F, was converted into ΔF/F = (F − F_baseline_)/F_baseline_.

### 2.4. Histology

As described previously, histological verification of viral expression was conducted [8,13,17]. In brief, the mice were first deeply anesthetized with an overdose of pentobarbital and then intracardially perfused with phosphate-buffered saline (PBS), followed by 4% paraformaldehyde in 0.1 M phosphate buffer. Then, the brains were post-fixed in 4% paraformaldehyde for 6 h after removal and transferred into 20% and 30% sucrose in PBS at 4 °C for cryoprotection until they sank to the bottom. After embedding and freezing, the brain samples were sectioned into 30 µm thick coronal slices using a freezing microtome (CM1950; Leica, Germany). The brain slices were rinsed three times in PBS, mounted on glass slides, and coverslipped with DAPI FluoromountG (Cat# 0100-20, Southern Biotech, Birmingham, AL, USA). Finally, fluorescence images were captured using a VS120 virtual microscopy slide scanning system (VS120, Olympus, Japan). We included only data from mice with confirmed AAV infection and optical fiber location.

### 2.5. Statistical Analysis

The data are presented as the mean ± standard error of the mean. Animal numbers (*n* = 5 per condition) were chosen based on our previous experience with similar experiments [14]. In this study, five mice were excluded from further analysis due to death, poor virus expression, misplaced optical fibers, and loose electrodes. Based on previous studies, one-way repeated-measures analysis of variance with Tukey post hoc tests were used for the pairwise comparison between groups [8,13,19,20]. Statistical analyses were conducted using Prism 10.3.0 software (GraphPad Software, San Diego, CA, USA), and *p* values < 0.05 were considered statistically significant.

## 3. Results

### 3.1. NAc DA Signal Dynamics in Response to 1.5%, 2.0%, and 2.5% Sevoflurane Anesthesia

**Figure 1:**
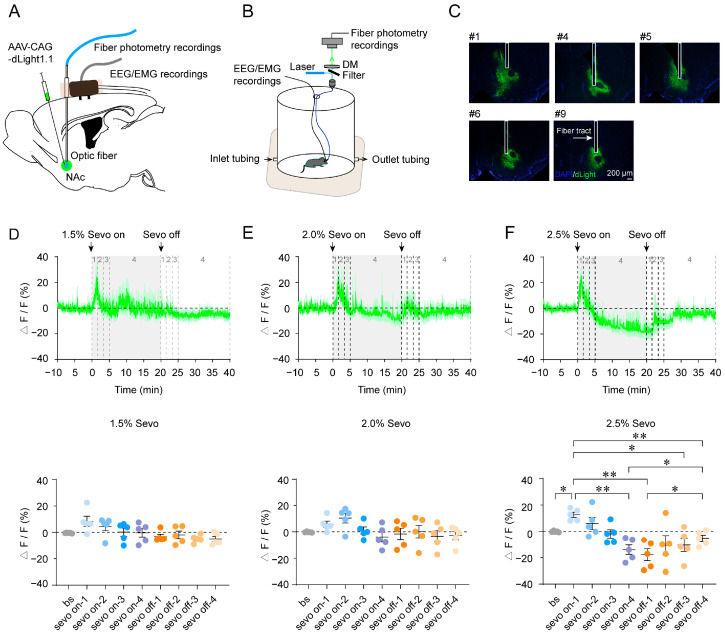
(**A**). Schematic of surgery for AAV-dLight1.1 injection into the NAc of C57BL/6J mice and implantation of optical fiber cannula and EEG/EMG electrode. (**B**). Schematic showing the setup for fiber photometry with simultaneous EEG/EMG recordings. (**C**). Expression of AAV-dLight1.1/DAPI in the NAc and track of the optic fiber implanted into the NAc (scale bar: 200 μm). (**D**–**F**). Top, time courses of mean (SEM) (shaded area) NAc dLight1.1 fluorescence signals following 1.5% (**D**), 2.0% (**E**), and 2.5% (**F**) sevoflurane administration. Bottom, average NAc dLight1.1 fluorescence signals 10 min before, 20 min during (sevo on-1: 0–100 s; sevo on-2:100–200 s; sevo on-3: 200–300 s; sevo on-4: 300–1200 s), and 20 min after (sevo off-1: 1200–1300 s; sevo off-2:1300–1400 s; sevo off-3: 1400–1500 s; sevo off-4: 1500–2400 s) 1.5%, 2.0%, and 2.5% sevoflurane administration. Data are shown as the mean (SEM). *n* = 5; * *p* < 0.05, ** *p* < 0.01. AAV: Adeno-associated virus; bs: baseline; DM: dichroic mirror; EEG: electroencephalogram; EMG: electromyogram; NAc: nucleus accumbens; Sevo: sevoflurane.

AAV-dLight1.1 was unilaterally injected into the NAc of C57BL/6J mice with implantations of optic fiber and EEG/EMG electrodes to examine whether the DA signal dynamics in the NAc correlate with states of consciousness across sevoflurane anesthesia stages (Figure 1A,B). In all mice, the expression of AAV-dLight1.1 fluorescence and the location of optic fiber in the NAc were confirmed by histology (Figure 1C). As shown in Figure 1D–F, after 1.5%, 2.0%, or 2.5% sevoflurane administration, the dLight1.1 fluorescence signals in the NAc rose rapidly during the initial 5 min of sevoflurane induction compared to the awake baseline state. Although mice were reported to lose consciousness under 2% sevoflurane exposure, NAc DA fluctuations did not differ significantly before, during, and after exposure to 1.5% or 2.0% sevoflurane (Figure 1D,E) [13,21]. However, under 2.5% sevoflurane exposure, NAc DA signals were significantly increased, reaching a peak around 100 s after sevoflurane exposure (baseline vs. sevo on-1, *p* = 0.0261; Figure 1F). Then, it gradually decreased and returned to baseline levels about 5 min after sevoflurane induction. In the maintenance phase (300–1200 s), NAc DA signal dynamics continued to decrease (sevo on-1 vs. sevo on-4, *p* = 0.0070; Figure 1F). After cessation of sevoflurane administration, the DA levels in the NAc gradually returned (sevo on-1 vs. sevo off-1, *p* = 0.0096; sevo on-1 vs. sevo off-3, *p* = 0.0490; sevo on-1 vs. sevo off-4, *p* = 0.0059; sevo on-4 vs. sevo off-4, *p* = 0.0340; sevo off-1 vs. sevo off-4, *p* = 0.0451; Figure 1F). Collectively, these findings indicate that NAc DA signal dynamics change in step with different stages of 2.5% sevofluraneanesthesia. 

### 3.2. NAc DA Signal Dynamics Across Sevoflurane Anesthesia Induction/Emergence Transitions

**Figure 2:**
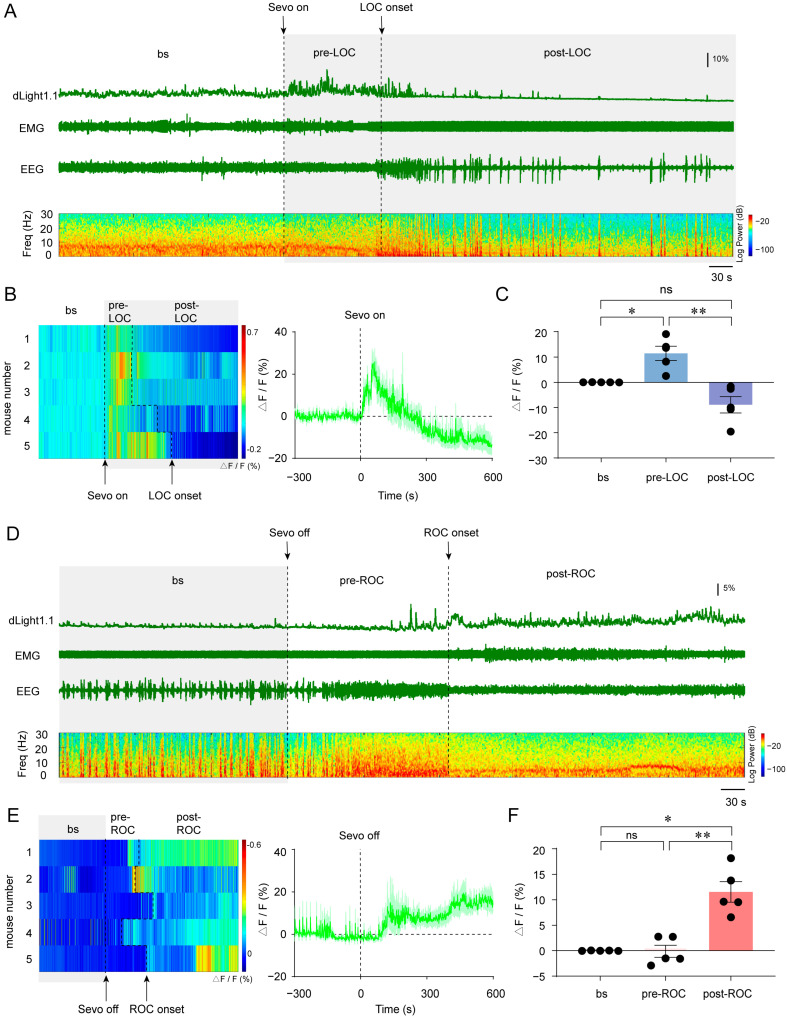
(**A**). Representative traces of NAc dLight1.1 fluorescent signal, EEG/EMG signal, and EEG power spectrograms during the induction of 2.5% sevoflurane anesthesia. (**B**). Heatmaps (left) and mean dLight1.1 fluorescent signal traces [SEM] (shaded area, right) for NAc DA during the LOC transition induced by 2.5% sevoflurane. (**C**). Quantification of NAc dLight1.1 signal changes in three consecutive time sections during the LOC transition. Data are presented as the mean (SEM); *n* = 5; * *p* < 0.05, ** *p* < 0.01. (**D**). Representative traces of NAc dLight1.1 fluorescent signals aligned to ROC state transitions. (**E**). Heatmaps (left) and mean dLight1.1 fluorescent signal traces [SEM] (shaded area, right) for NAc DA during the ROC transition during emergence from 2.5% sevoflurane anesthesia. (**F**). Quantification of NAc dLight1.1 signal changes in three consecutive time sections during the ROC transition. Data are presented as the mean (SEM); *n* = 5; * *p* < 0.05, ** *p* < 0.01. bs: baseline; EEG: electroencephalogram; EMG: electromyogram; Freq: frequency; LOC: loss of consciousness; ROC: recovery of consciousness; Sevo: sevoflurane.

Next, we analyzed real-time DA signal dynamics in the NAc during the induction and emergence periods of 2.5% sevoflurane anesthesia. During the induction phase (Figure 2A,B), the DA levels in the NAc were pronouncedly elevated during the pre-LOC period (pre-anesthesia baseline vs. pre-LOC, *p* = 0.0329; Figure 2C). The NAc DA levels significantly declined after LOC compared with the pre-LOC period (pre-LOC vs. post-LOC, *p* = 0.0094; Figure 2C). For the emergence phase (Figure 2D,E), as illustrated in Figure 2F, there was no statistically significant change in the DA levels between the anesthesia baseline and the pre-ROC period (anesthesia baseline vs. pre-ROC, *p* = 0.9957). However, the NAc DA levels showed a conspicuous increase during the initial period after ROC (anesthesia baseline vs. post-ROC, *p* = 0.0103; pre-ROC vs. post-ROC, *p* = 0.0086; Figure 2F). Taken together, these findings demonstrate that the NAc DA signal dynamics are correlated with consciousness transition across sevoflurane anesthesia induction and emergence.

### 3.3. NAc DA Fluctuations Correlate with Burst Suppression Events

**Figure 3:**
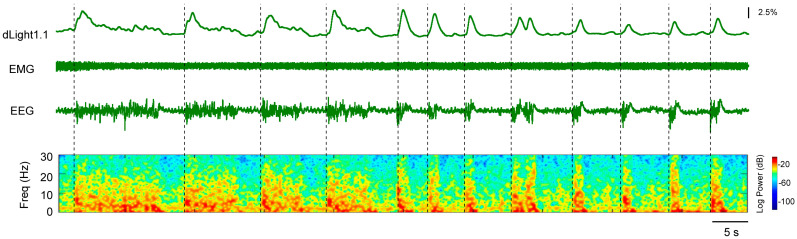
Representative traces of NAc dLight1.1 fluorescent signal, EEG/EMG signal, and EEG power spectrograms during burst suppression events induced by 2.5% sevoflurane anesthesia. EEG: electroencephalogram; EMG: electromyogram; Freq: frequency.

Next, we analyzed the real-time NAc dLight1.1 signal dynamics during the maintenance phase of 2.5% sevoflurane anesthesia. Surprisingly, we observed that DA release in the NAc correlated with burst suppression oscillation events (a deep anesthesia state). The DA signals rapidly reached a peak when the burst wave was initiated and then gradually attenuated, indicating a positive correlation with the onset of the burst wave during burst suppression oscillations (Figure 3). Conversely, the DA signals showed no obvious fluctuation when a suppression wave was exhibited. Collectively, these findings indicate that the release of DA neurotransmitters in the NAc correlates with burst suppression events during sevoflurane anesthesia.

## 4. Discussion

In this study, by employing a DA sensor and simultaneous EEG/EMG recordings, we observed that a conspicuous increase in NAc DA release preceded the onset of LOC and an obvious increase during the ROC transition. Surprisingly, we revealed that the release of the NAc DA neurotransmitter was positively associated with sevoflurane-induced agitation during induction and the onset of burst waves during burst suppression oscillations. Collectively, this evidence suggests that NAc DA signal dynamics correlate with different states of consciousness across sevoflurane anesthesia stages.

Numerous studies have confirmed the important regulatory role of the central DAergic system in GA modulation. However, previous studies tended to focus on the DAergic neurons/neural circuits and DA receptors [3,13,22,23]. While these are undoubtedly pivotal, it is also essential to recognize that elucidating neurotransmitter dynamics in specific brain areas is essential to comprehensively understand the neural mechanism of GA. In this study, for the first time, we focus on the DA neurotransmitter itself, exploring the role of NAc DA dynamics throughout sevoflurane anesthesia and offering a new perspective on the neural mechanism of GA.

Instead of a direct decrease in the activities of DAergic neurons and NAc DA D1 receptor-expressing neurons after sevoflurane administration [13,24], we observed a rapid increase in NAc DA signal dynamics accompanied by behavioral hyperactivity during the initial period of sevoflurane induction. This phenomenon may be attributed to the significant role of NAc DA in reward, motivation, and arousal [17,25,26]. It has also been reported that aversive stimuli increase DA levels in the NAc [27]. Thus, we speculated that the rapid rise of DA fluctuations may be associated with the state of paradoxical excitation (also called anesthetic-induced agitation) in the GA induction period, which is not uncommon in clinical practice and animal studies [28,29,30,31]. However, the role of NAc DA signal dynamics in the modulation of paradoxical excitation during anesthesia induction requires further investigation. During sevoflurane emergence, we also observed a rapid increase in the NAc DA level during the initial period after ROC. This indicates that the NAc DA signal is important for maintaining an awake state during the early stage of consciousness recovery. Exploring the neural mechanism of NAc DA signal dynamics may also enhance our understanding of the mechanism of consciousness transition under GA.

Burst suppression is an EEG signal marked by alternating high-voltage activity (the burst waves) and isoelectricity (the suppression) of EEG signals [32]. Recent research has revealed that the synchronous activity of pyramidal neurons and cortical glutamatergic neurons in the cerebral cortex is the primary cause of burst suppression [33,34]. Additionally, some studies have suggested that the striatum exhibits a strong correlation with burst suppression during GA [35]. Here, for the first time, we report that DA levels in the ventral striatum exhibited regular synchronous changes during burst suppression events. Using high spatial and temporal resolution, our findings offer new insights for future investigations to further elucidate the mechanisms of burst suppression oscillations associated with a deep anesthesia state and provide potential theoretical support for monitoring anesthesia depth.

Despite its strengths, this study has some limitations. One limitation of this study is that we focused exclusively on DA in the NAc; however, the correlations of other monoamine neurotransmitters (e.g., norepinephrine and serotonin) and other brain regions releasing DA (e.g., dorsal striatum, medial prefrontal cortex, and hypothalamus) with states of consciousness under sevoflurane anesthesia also deserve further investigation. In addition to ventral tegmental area DAergic neurons, cholinergic interneurons in the NAc and projections from the basolateral amygdala trigger NAc DA release [11]. It is worth exploring whether NAc cholinergic interneurons and other afferent projections of the NAc are involved in regulating states of consciousness under sevoflurane anesthesia and their interaction with the DAergic system.

At present, clinical anesthesiologists mainly relied on an EEG/Bispectral index monitor to determine anesthesia depth. Also, there is a lack of specific drugs that can rapidly promote the recovery from GA. Exploring the neural mechanism of GA provides an important theoretical basis for developing new monitoring methods/specific drugs and preventing perioperative complications.

## 5. Conclusions

In conclusion, these findings enhance our understanding of the neural mechanisms of GA. Our findings revealed that the NAc DA neurotransmitter signal dynamics correlate with different states of consciousness throughout sevoflurane anesthesia and offer a potential method for optimizing anesthesia management.

## Data Availability

The data and analyses used in this study can be made available from the corresponding author upon reasonable request.
